# CXL-1020, a Novel Nitroxyl (HNO) Prodrug, Is More Effective than Milrinone in Models of Diastolic Dysfunction—A Cardiovascular Therapeutic: An Efficacy and Safety Study in the Rat

**DOI:** 10.3389/fphys.2017.00894

**Published:** 2017-11-10

**Authors:** Steve R. Roof, Yukie Ueyama, Reza Mazhari, Robert L. Hamlin, J. Craig Hartman, Mark T. Ziolo, John E. Reardon, Carlos L. del Rio

**Affiliations:** ^1^QTest Labs, Columbus, OH, United States; ^2^Cardioxyl Pharmaceuticals, Chapel Hill, NC, United States; ^3^Ohio State University Columbus, Columbus, OH, United States

**Keywords:** nitroxyl, CXL-1020, cardiac diastolic dysfunction, cardiovascular therapeutic

## Abstract

The nitroxyl (HNO) prodrug, CXL-1020, induces vasorelaxation and improves cardiac function in canine models and patients with systolic heart failure (HF). HNO's unique mechanism of action may be applicable to a broader subset of cardiac patients. This study investigated the load-independent safety and efficacy of CXL-1020 in two rodent (rat) models of diastolic heart failure and explored potential drug interactions with common HF background therapies. *In vivo* left-ventricular hemodynamics/pressure-volume relationships assessed before/during a 30 min IV infusion of CXL-1020 demonstrated acute load-independent positive inotropic, lusitropic, and vasodilatory effects in normal rats. In rats with only diastolic dysfunction due to bilateral renal wrapping (RW) or pronounced diastolic and mild systolic dysfunction due to 4 weeks of chronic isoproterenol exposure (ISO), CXL-1020 attenuated the elevated LV filling pressures, improved the end diastolic pressure volume relationship, and accelerated relaxation. CXL-1020 facilitated Ca^2+^ re-uptake and enhanced myocyte relaxation in isolated cardiomyocytes from ISO rats. Compared to milrinone, CXL-1020 more effectively improved Ca^2+^ reuptake in ISO rats without concomitant chronotropy, and did not enhance Ca^2+^ entry via L-type Ca^2+^ channels nor increase myocardial arrhythmias/ectopic activity. Acute-therapy with CXL-1020 improved ventricular relaxation and Ca^2+^ cycling, in the setting of chronic induced diastolic dysfunction. CXL-1020's lusitropic effects were greater than those seen with the cAMP-dependent agent milrinone, and unlike milrinone it did not produce chronotropy or increased ectopy. HNO is a promising new potential therapy for both systolic and diastolic heart failure.

## Introduction

Acute decompensated heart failure (ADHF) is the leading cause of hospitalization in the United States with ~1 million hospital admissions per year (Feldman et al., 2008). Heart failure is a complex syndrome, characterized by the inability of the heart to maintain adequate cardiac output without elevated intracardiac filling pressures, leading to pulmonary and peripheral fluid retention and the typical clinical findings of fatigue, dyspnea and edema. During episodes of ADHF, the principles of care include decreasing preload and afterload, and augmenting cardiac contractility. Patients with heart failure receive diuretics such as furosemide, vasodilators such as nitrates, and as needed, intravenous inodilators/inotropes such as, milrinone (MIL), and dobutamine (DOB). The goals of in-hospital therapy are to relieve acute patient discomfort (primarily dyspnea) and restore baseline hemodynamics to the extent possible for these often critically ill patients. Despite current pharmacologic strategies, mortality (both in-hospital and post-discharge) and readmission for ADHF remains high. While existing inodilators and inotropes can improve acute hemodynamics, they confer deleterious side effects and increase mortality (Packer et al., 1991, 2013; Cuffe et al., 2002). Safer, effective inodilators are needed.

Nitroxyl (HNO), has been shown to improve cardiac function in canine models of systolic heart failure by directly enhancing inotropy and lusitropy, and by venous and arterial vasodilation (pre-/afterload reduction) (Paolocci et al., 2003, 2007; Sabbah et al., 2013) without chronotropy, arrhythmogenicity, or an increase in energetic demand. In clinical studies, the HNO prodrug, CXL-1020, produced an attractive hemodynamic profile in patients with decompensated systolic heart failure, demonstrating the potential for HNO to be a useful treatment for ADHF (Sabbah et al., 2013). *In vitro* studies have elucidated the molecular mechanisms of HNO's pleiotropic effects in the heart and peripheral vasculature. HNO vasorelaxation is mediated by activation of soluble guanylate cyclase (Irvine et al., 2007; Paolocci et al., 2007). In addition, HNO enhances cardiac performance and ventriculo-arterial coupling (VAC) by modulating the excitation–contraction coupling (ECC) process and the function of myofilament proteins. HNO enhances both sarcoplasmic reticulum (SR) Ca^2+^ uptake through the SERCA pump (Lancel et al., 2009; Sivakumaran et al., 2013) and SR fractional Ca^2+^ release via a direct thiol sensitization of the type 2 ryanodine receptor (RyR2) (Cheong et al., 2005; Tocchetti et al., 2007), enhancing systolic Ca^2+^ transients without altering basal cardiac I_Ca_ current/Ca^2+^ entry (Kohr et al., 2010), increasing diastolic Ca^2+^ or causing SR Ca^2+^ overload.

HNO also increases myofilament Ca^2+^ sensitivity and the force of contraction through the formation of disulfide bonds in myofilament proteins (Redfield et al., 2003). The cardiac activity of HNO is neither cGMP- or cAMP-dependent and is readily distinguished from the cAMP-dependent activity of MIL and DOB.

The animal and clinical studies described above were conducted in hearts with depressed systolic function (reduced ejection fraction; EF). However, ~50% of HF patients have heart failure where EF is preserved (HFpEF) (Redfield et al., 2003; Halley et al., 2011). The efficacy of HNO has yet to be studied in this setting where diastolic dysfunction is often observed, combining abnormal VAC, delay in active relaxation, increased diastolic chamber stiffening, and reduced cardiac reserve (Kitzman et al., 1991; Borlaug and Kass, 2008; Borlaug, 2014). The present study tested this in two well-established rat models of cardiac diastolic dysfunction: chronic hypertension secondary to renal-wrapping (RW) and chronic beta-adrenergic stimulation (ISO). The acute efficacy and safety of HNO was compared to milrinone, and when administered in combination with clinically established background therapies; the β-adrenoreceptor (β-AR) antagonist, metoprolol, and the angiotensin-converting enzyme (ACE) inhibitor, enalapril. HNO (donated by CXL-1020) improved cardiac inotropy/lusitropy and Ca^2+^ handling in rats with abnormal relaxation, without inducing chronotropy or increasing background ectopy, and with reduced LV pressure-volume area (PVA) (an estimate of total mechanical energy requirement; a correlate of myocardial oxygen consumption) (Wannenburg et al., 1992).

## Materials and methods

### Animals models

Adult male Sprague-Dawley rats (250–350 g; Harlan Laboratories, Indianapolis, IN) were used in these experiments. Animal care and experimental protocols were approved by the Animal Care and Use Committee of QTest Labs.

### Induction of cardiac dysfunction

Alzet mini-osmotic pumps (Alzet, Cupertino, CA; model 2004), filled with isoproterenol (ISO) to deliver 1 mg/kg/day for 4 weeks at a rate of 0.25 μL/h, were implanted in anesthetized rats (~45/~5 mg/kg) via a ~1 cm in a mid-scapulae subcutaneous pocket to the manufacturer's instructions, as previously described (Takeshita et al., 2008; Roof et al., 2016). Prior to terminal experiments, rats were anesthetized and the ISO pump was harvested 1 h before a data collection as described below. Renal wrap (RW) rats were anesthetized rats (~45/~5 mg/kg), a midline laparotomy was performed, both kidneys were isolated, freed from the renal capsule, and a figure-8 knot using 2-0 silk suture will be placed around each kidney. After surgical recovery, the animals were followed for 5 weeks.

### Echocardiography

Anesthetized rats (ketamine/xylazine: 45/5 mg/kg) were positioned in right-lateral recumbence and transthoracic 2D-guided short-axis M-mode examinations were assessed at the mid-papillary level to evaluate left ventricular ejection fraction via echocardiography (Phillips HD11xe). Doppler echo was used to assess the ratio of the early (E) to late (A) ventricular filling velocities (E/A ratio) at the level of the mitral valve from an apical four-chamber view on rats positioned in dorsal recumbence.

### Surgical procedures and instrumentation

Each rat was intraperitoneally (IP) anesthetized with sodium pentobarbital (50–90 mg/kg), positioned in dorsal recumbence, endotracheally intubated and ventilated (~90 breaths/min, ~2.5 mL tidal volume with 95%/5% (O_2_/CO_2_) with an adjustable small animal ventilator (Harvard Apparatus). Anesthesia was maintained with a continuous sodium pentobarbital intravenous (IV) infusion (4 mg/kg/h) via an indwelling catheter placed in a peripheral vein, until completion of the experiment. A micromanometer catheter (Millar Instruments—SPR-407) was inserted into the femoral artery to measure arterial pressure. For left ventricular (LV) mechano-energetic evaluation, a 2F high-fidelity conductance/micromanometer catheter (Millar Instruments—SPR-869) was advanced retrograde across the aortic valve and into the LV chamber by way of the right carotid artery in order to simultaneously determine left-ventricular pressure and volume (via conductivity); despite calibration in blood and parallel-conductance adjustments, volumes are expressed as relative volume units (RVU). A balloon catheter was placed and advanced into the inferior vena cava through the left femoral vein. At specific timepoints during the experiment, this balloon was inflated to acutely decrease myocardial preload in order to generate a family of pressure-volume curves/loops. The resulting left-ventricular pressure and volume data were analyzed offline (IOX/ECG Auto; EMKA Technologies) in order to generate relationships representing the contractile and energetic state of the myocardium. In all cases, hemodynamic stabilization was allowed for at least 15 min prior to the start of the experiments.

### *In vivo* cardiovascular assessments

The cardiovascular effects of HNO (CXL-1020, 100 μg/kg/min) were compared with volume/vehicle controls (7% Captisol, CyDex, Lawrence, KS), both with and without intravascular volume expansion with Hextend (HEX; Braun, Irvine, CA). In addition, load-independent functional effects of HNO were compared (at matched blood pressure reductions) to those of the clinically-used nitric oxide donor sodium nitroprusside (SNP, 25 μg/kg/min, IV), both with and without concomitant restoration of systemic pressures via phenylephrine (PE, 5 μg/kg/min, IV).

The cardiovascular efficacy and safety of CXL-1020, was compared to the phosphodiesterase 3A (PDE3A) inhibitor milrinone (MIL, 10 μg/kg/min, IV) in the setting of normal and abnormal (ISO, RW) cardiac function. Pro-arrhythmia was quantified by the number of LV ectopic beats per minute based on the LV-pressure waveform.

The interactions of HNO were evaluated in combination with the β –AR antagonist, metoprolol (MET, 1 mg/kg /h, IV) and the ACE inhibitor enalaprilat (ENA, 1 mg/kg/h, IV). The effects of HNO and MIL were also compared in the setting of L-type Ca^2+^ channel (LTCC) blockade with verapamil (VER, 0.3 mg/kg bolus).

LV ectopic beats were analyzed using ecgAuto from the LV pressure wave form.

### Cardiomyocyte isolation

Ventricular myocytes were isolated from anesthetized rats following an IP injection of ketamine/xylazine (~45/~5 mg/kg). The heart was cannulated and hung on a Langendorff apparatus. It was then perfused with Ca^2+^ free tyrode solution (consisted of (in mmol/L): 140 NaCl, 4 KCl, 1 MgCl_2_, 10 glucose, 5 HEPES, pH 7.4 adjusted with NaOH or HCl) for 4 min. The solution was then switched to a tyrode solution containing collagenase Type II (59 mg/100 mL) (Worthington, Lakewood, NJ), hyaluronidase (2.4 mg/100 mL) (Sigma, H-3506, St Louis, MO), and protease (3.5 mg/100 mL) (Sigma, P-5147, St Louis, MO). After 30 min, the heart was taken off the Langendorff, the ventricles minced while in a petri dish filled with 0 Ca^2+^ Tyrode solution via tissue scissors, and myocytes were dissociated by trituration with a transfer pipette. Subsequently the myocytes were filtered through a 200 um nylon mesh filter, briefly centrifuged for ~5 s, and resuspended in tyrode solution containing 200 μmol/L Ca^2+^. Myocytes were used within 6 h of isolation, *as* previously described (Roof et al., 2013).

### Measurement of myocyte Ca^2+^ transients and shortening

Ca^2+^ transient and shortening measurements were performed at room temperature with tyrode solution with 1 mmol/L CaCl_2_ (2 mmol/L CaCl_2_ for canine), as previously described (Roof et al., 2011, 2013). Briefly, myocytes were loaded at room temperature with Fluo-4 AM (10 μmol/L, Molecular Probes, Eugene, OR) for 30 min. An additional 30 min were allowed for intracellular de-esterification. The instrumentation used for cell fluorescence measurements was a Cairn Research Limited (Faversham, UK) epifluorescence system. [Ca^2+^]_i_ was measured by Fluo-4 epifluorescence with excitation at 480 ± 20 nm and emission at 535 ± 25 nm. The illumination field was restricted to collect the emission of a single cell. Data is expressed as ΔF/F_0_, where F is the fluorescence intensity and F_0_ is the intensity at rest. Data for cell shortening was collected using a video edge detection system (Crescent Electronics). Myocytes were stimulated at 1 Hz via platinum electrodes connected to a Grass Telefactor S48 stimulator (West Warwick, RI). As Mil alone did not elicit a functional effect (as previously demonstrated; Raffaeli et al., 1989), following baseline, all cells were perfused with a low dose of ISO (0.1 nM) to increase intracellular cAMP to enable MIL to have an effect, but yet, 0.1 nM ISO did not have a significant function effect alone. Both CXL-1020 and Mil solutions also contained 0.1 nM ISO. The 0.1 nM ISO steady state was used as the baseline.

### Statistical analysis

Differences between group were evaluated (Prism 6; GraphPad Software, La Jolla California USA) via either paired/unpaired Student's *t*-tests or by one-way ANOVA with *post-hoc* analyses via Bonferroni ‘s multiple-comparison test. In all cases, statistical assumptions were verified, and *P* < 0.05 was considered statistically significant *a priori*.

## Results

### CXL-1020 improves hemodynamics and cardiac function in normal rats and is effective when combined with common clinical HF medications

**Figure 1:**
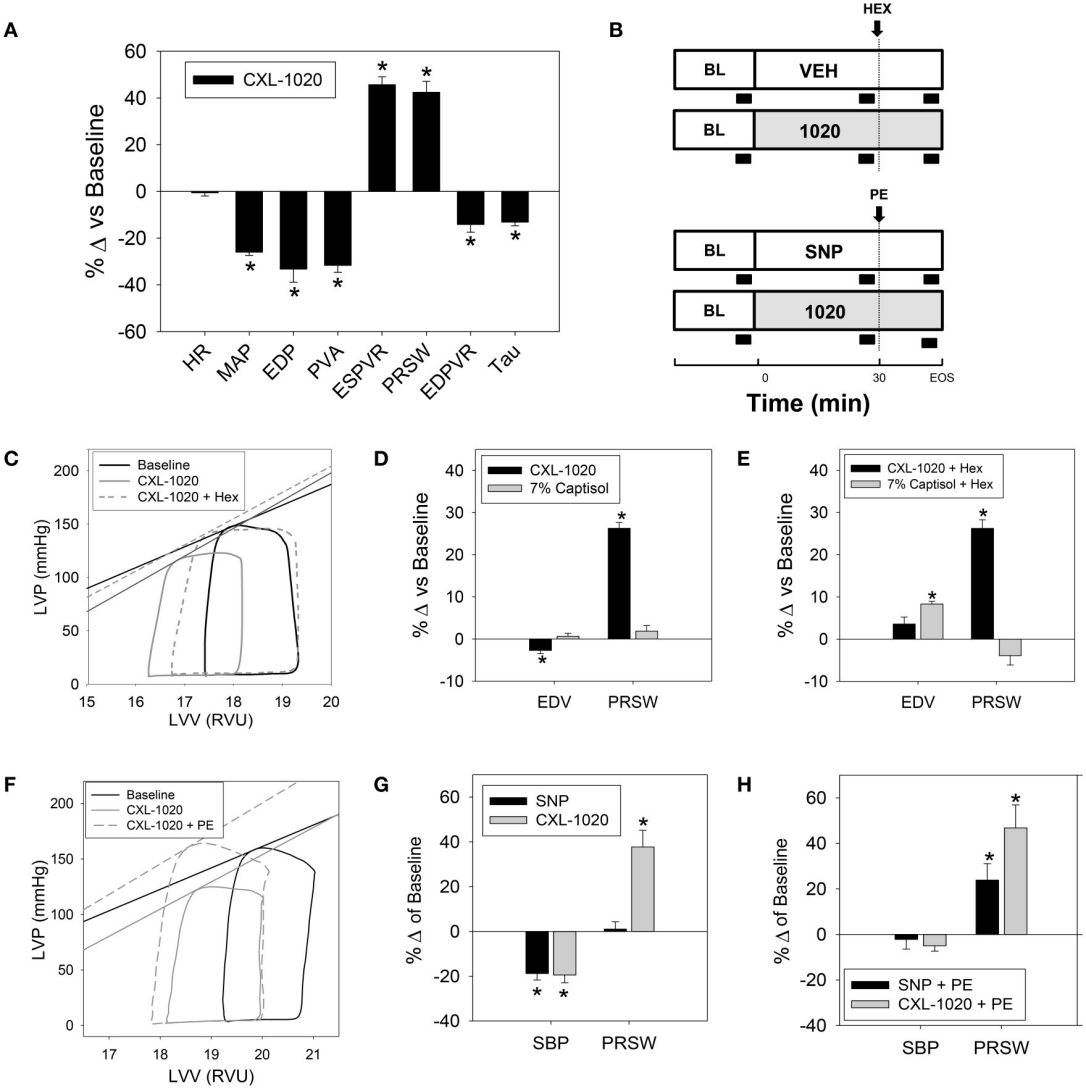
Effects of CXL-1020 on systemic, energetic, and left-ventricular hemodynamic indices in instrumented normal anesthetized rats. **(A)** Percent change vs. baseline following 30 min of continuous IV infusion of CXL-1020 (100 μg/kg/min, *n* = 31). **(B)** Schematic of the experimental design with black block indicating data acquisition. **(C)** Representative PV loops and % change from baseline comparing CXL-1020 (*n* = 7) to volume matched vehicle control (7% Captisol, *n* = 8) **(D)** before and **(E)** during volume expansion with Hextend (~1–1.5 mL). **(F)** Representative PV loops and % change from baseline comparing CXL-1020 (*n* = 7) to SNP (25 μg/kg/min, *n* = 6) **(G)** before and **(H)** during afterload restoration with PE (5 μg/kg/min). ^*^P < 0.05 vs. corresponding baseline.

Throughout this study, 31 instrumented normal rats received CXL-1020 (100 μg/kg/min for 30 min); a dose determined previously to be effective (Sabbah et al., 2013). The aggregate results for these experiments are summarized in Table 1 (vehicle control shown in Table 2). CXL-1020 significantly decreased mean arterial pressure (MAP) as well as left ventricular end-systolic (LV-ESP) and end-diastolic (LV-EDP) filling pressures (Table 1 and Figure 1A). These hemodynamic changes were not accompanied by heart rate (HR) increases. The slopes of the end-systolic pressure-volume relationship (ESPVR) and the preload recruitable stroke work (PRSW), two load-independent indices of contractility, were increased (Table 1 and Figure 1A). CXL-1020 decreased pressure volume area (PVA), suggesting a reduction in the estimated total mechanical energy requirement. The linear slope of the end diastolic pressure-volume relationship (EDPVR) and the time-constant of myocardial relaxation (Tau, from the LV pressure) both declined. While the above results reflect positive inotropy, the declines in preload and afterload may affect the interpretation. Therefore, additional studies were performed in which CXL-1020 preload reduction was restored by volume expansion with hextend (HEX) (see schematic of the experimental design with black block indicating data acquisition in Figure 1B and representative in Figure 1C). Demonstrated in Figure 1D (without HEX) and Figure 1E (with HEX), restoring of preload did not alter the CXL-1020-mediated increase in PRSW compared to controls. Moreover, when the arterial pressure reductions were matched using sodium nitroprusside (SNP) (Table 3, representative in Figure 1F and graphed in Figure 1G), and then antagonized by phenylephrine (PE) (Figure 1H), there was still a significant rise in PRSW compared to controls; confirming the load independence of the improvements in systolic performance and the reliability of PRSW as a readout for systolic function.

**Table 1:** Cardiovascular activity of CXL-1020 in instrumented anesthetized normal rats.

**Parameters**	**Normal**		
	**Baseline**	**CXL-1020**	**% Δ from baseline**
HR (bpm)	368 ± 6	366 ± 6	−1 ± 1
MAP (mmHg)	122 ± 2	94 ± 3^*^	−26 ± 1
LV—ESP (mmHg)	129 ± 3	95 ± 4^*^	−25 ± 3
LV—EDP (mmHg)	5.4 ± 0.5	4.4 ± 0.5^*^	−33 ± 6
LV—EDV (RVU)	19.3 ± 0.5	18.8 ± 0.5^*^	−4 ± 1
SW (mmHg * RVU ^*^ 10^3^)	2.3 ± 0.2	1.8 ± 0.1^*^	−24 ± 3
PVA (mmHg * RVU ^*^ 10^3^)	2.8 ± 0.2	2.0 ± 0.1^*^	−32 ± 3
ESPVR (mmHg/RVU)	22.9 ± 1.7	28.2 ± 1.4^*^	46 ± 3
PRSW (mmHg)	45 ± 2	61 ± 3^*^	42 ± 5
EDPVR (mmHg/RVU)	1.8 ± 0.1	1.5 ± 0.1^*^	−14 ± 3
LV—Tau (ms)	8.4 ± 0.2	7.6 ± 0.2^*^	−13 ± 2

*Changes in systemic and LV hemodynamics indices, and LV mechanical parameters were measured at baseline, and following 30 min of continuous IV infusion of CXL-1020 (100 μg/kg/min, n = 31). Values are mean ± standard error of the mean (SEM)*.

* *P < 0.05 vs. baseline*.

**Table 2:** Cardiovascular activity of vehicle control (7% Captisol) in instrumented anesthetized normal rats.

**Parameters**	**Normal**		
	**Baseline**	**7% Captisol**	**% Δ from baseline**
HR (bpm)	323 ± 12	317 ± 11	−2 ± 1
MAP (mmHg)	110 ± 4	115 ± 4	4 ± 2
LV—ESP (mmHg)	115 ± 5	119 ± 5	5 ± 3
LV—EDP (mmHg)	4.3 ± 0.7	5.0 ± 0.9	21 ± 16
LV—EDV (RVU)	19.4 ± 0.9	19.6 ± 1.0	1 ± 1
SW (mmHg ^*^ RVU ^*^ 10^3^)	2.4 ± 0.2	2.5 ± 0.2	2 ± 5
PVA (mmHg ^*^ RVU ^*^ 10^3^)	2.8 ± 0.2	2.8 ± 0.2	0 ± 5
ESPVR (mmHg/RVU)	16.5 ± 1.2	16.6 ± 1.3	1 ± 3
PRSW (mmHg)	43 ± 1	43 ± 1	2 ± 1
EDPVR (mmHg/RVU)	0.9 ± 0.1	1.0 ± 0.1	13 ± 11
LV—Tau (ms)	9.3 ± 0.3	9.4 ± 0.4	0 ± 2

*Changes in systemic and LV hemodynamics indices, and LV mechanical and energetic parameters were measured at baseline, and following at least 30 min of continuous IV infusion of 7% Captisol (n = 8). Values are mean ± standard error of the mean*.

**Table 3:** Effects of clinically established drugs on estimated mechanical energy and LV hemodynamics indices in instrumented anesthetized normal rats.

**Parameters**	**Drug**	**Normal**		
		**Baseline**	**Drug**	**% Δ frombaseline**
HR (bpm)	SNP	385 ± 10	394 ± 8	3 ± 2
	DOB	354 ± 9	436 ± 16^*^	24 ± 5
MAP (mmHg)	SNP	116 ± 7	80 ± 7^*^	−32 ± 3
	DOB	114 ± 5	114 ± 5	0 ± 4
PVA (mmHg ^*^ RVU ^*^ 10^3^)	SNP	2.7 ± 0.6	2.9 ± 0.6^*^	−22 ± 10
	DOB	3.9 ± 0.6	5.9 ± 1.0^*^	54 ± 19
PRSW (mmHg)	SNP	75 ± 13	76 ± 19	−4 ± 7
	DOB	48 ± 5	99 ± 13^*^	110 ± 19
EDPVR (mmHg/RVU)	SNP	1.8 ± 0.4	1.6 ± 0.8	0 ± 17
	DOB	2.6 ± 0.6	1.8 ± 0.4^*^	−30 ± 5

*Summary data as values are mean ± standard error of the mean and % change from baseline of sodium nitroprusside (SNP) (25 μg/kg/min, n = 6), dobutamine (DOB) (5 μg/kg/min, n = 7)*.

* *P < 0.05 vs. corresponding baseline*.

We investigated the pharmacodynamic drug-drug interactions of CXL-1020 combined with other cardioactive drugs commonly used in patients with HF. The cardiovascular profile obtained by combining CXL-1020 and ACE-inhibition with enalapril (ENA) was similar to CXL-1020 alone except for a mildly enhanced reduction in MAP (Table 4 and Figure 2A). Consistent with its β-AR-independent mechanism of action, the inotropic and lusitropic effects of CXL-1020 were retained when combined with β-AR blockade (metoprolol, MET, Table 4, Figure 2B). The arterial blood pressure, inotropic response and PVA of CXL-1020 + DOB (Table 4) was a composite of the individual profiles of CXL-1020 (Table 1) and DOB (Table 3), illustrating their unique and independent mechanisms of action and confirming previous observations (Paolocci et al., 2003).

**Table 4:** Effects of clinically established drugs and CXL-1020 when administered in combination on estimated mechanical energy and LV hemodynamics indices in instrumented anesthetized normal rats.

**Parameters**	**Drug**	**Normal**		
		**Baseline**	**Drug**	**% Δ from baseline**
MAP (mmHg)	CXL-1020	123 ± 4	92 ± 9^*^	−26 ± 6
	CXL-1020 + ENA	–	69 ± 9	−44 ± 7^#^
	CXL-1020	114 ± 5	85 ± 5^*^	−26 ± 4
	CXL-1020 + MET	–	67 ± 5	−41 ± 5^#^
	CXL-1020 + DOB	–	81 ± 6	−29 ± 5
PVA (mmHg ^*^ RVU ^*^ 10^3^)	CXL-1020	2.7 ± 0.5	1.9 ± 0.3^*^	−28 ± 6
	CXL-1020 + ENA	–	1.7 ± 0.3^*^	−33 ± 5
	CXL-1020	3.9 ± 0.6	2.5 ± 0.4^*^	−37 ± 7
	CXL-1020 + MET	–	2.4 ± 0.3^*^	−43 ± 6
	CXL-1020 + DOB	–	4.5 ± 0.7^*^	24 ± 16^#^
PRSW (mmHg)	CXL-1020	56 ± 11	79 ± 8	56 ± 10
	CXL-1020 + ENA	–	76 ± 5	56 ± 20
	CXL-1020	48 ± 5	73 ± 11	51 ± 15
	CXL-1020 + MET	–	64 ± 6	39 ± 19
	CXL-1020 + DOB	–	114 ± 9	125 ± 20^#^

*Summary data as % change from baseline of CXL-1020 administered with an ACE inhibitor, (ENA, 1 mg/kg/h, n = 5), β-AR blocker, MET (1 mg/kg/h, n = 7), and β-AR 1 agonist, DOB (5 μg/kg/min, n = 7)*.

* *P < 0.05 vs. corresponding baseline*,

# *P < 0.05 vs. corresponding CXL-1020 alone*.

**Figure 2:**
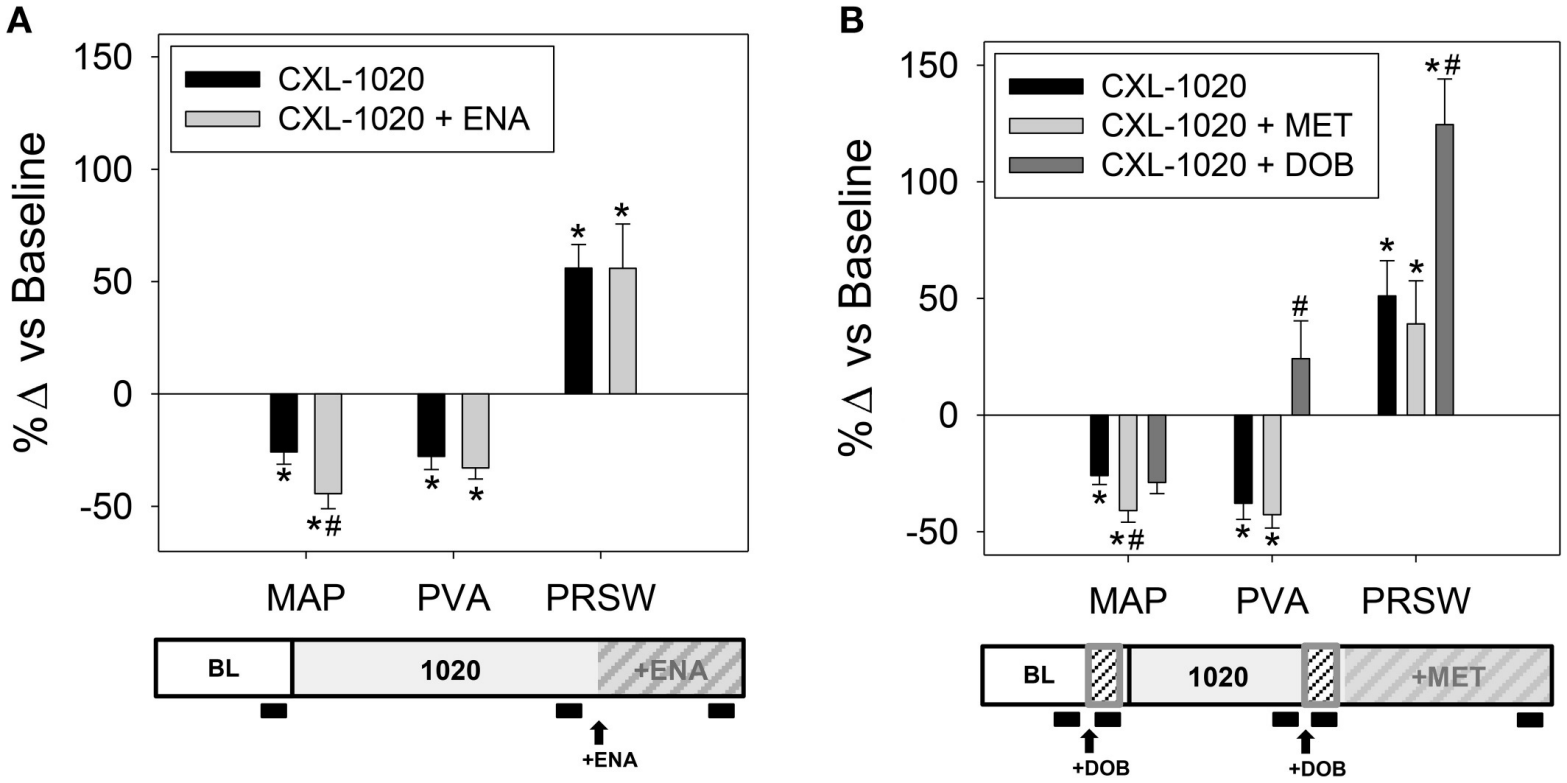
Effects of clinically established drugs and CXL-1020 when administered in combination on estimated mechanical energy and LV hemodynamics indices in instrumented anesthetized normal rats. Summary data as % change from baseline of CXL-1020 administered with an **(A)** ACE inhibitor, (ENA, 1 mg/kg/hr, *n* = 5), **(B)** β-AR blocker, MET (1 mg/kg/h, *n* = 7), and β-AR 1 agonist, DOB (5 μg/kg/min, *n* = 7). Experimental design schematic with black block indicating data acquisition is below each corresponding graph. ^*^P < 0.05 vs. corresponding baseline, ^#^*P* < 0.05 vs. corresponding CXL-1020 alone.

### CXL-1020 inotropic and lusitropic efficacy is enhanced in a setting of cardiac dysfunction

The activity of CXL-1020 was investigated in two models of cardiac dysfunction: chronic renoprival hypertension secondary to bilateral renal wrapping (RW) and chronic β-AR stimulation (ISO).

**Figure 3:**
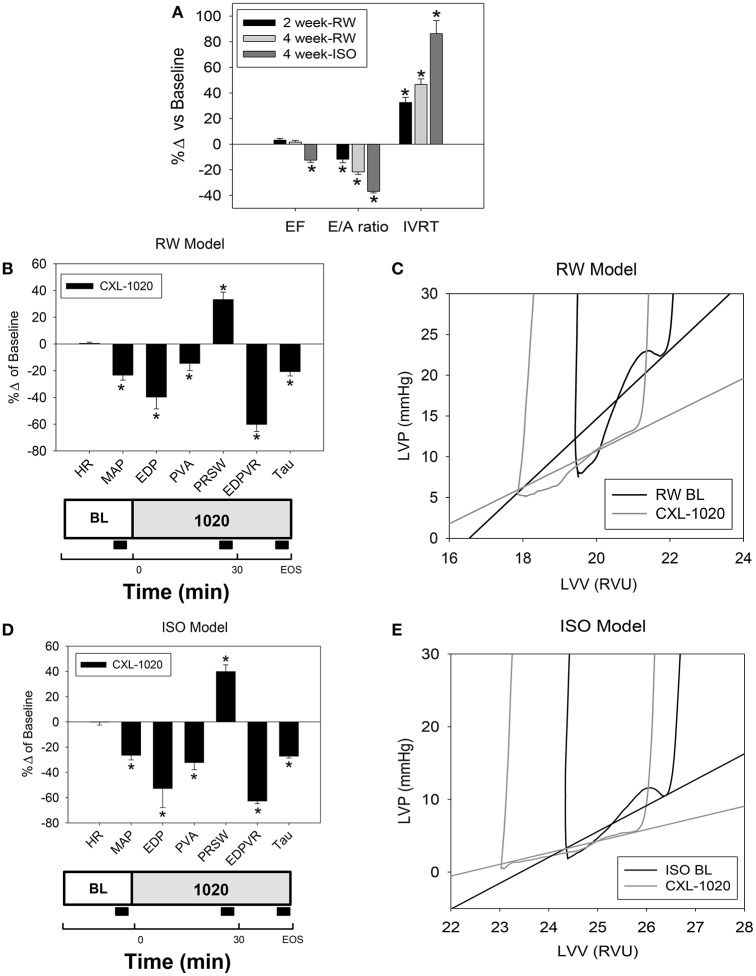
Effect of CXL-1020 on systemic and LV hemodynamics indices in instrumented anesthetized RW and ISO rat models. **(A)** Echocardiographic evaluation on systolic and diastolic indices in RW (*n* = 15) and ISO rats (*n* = 6). **(B)** Cardiovascular profiles of CXL-1020 (100 μg/kg/min, *n* = 8) as shown as % change from baseline following 5 weeks of renal wrapping. **(C)** Representative single PV loop of the diastolic portion. **(D)** Cardiovascular profile of CXL-1020 (100 μg/kg/min, *n* = 6) as shown as % change from baseline following 4 weeks of chronic ISO. **(E)** Representative single PV loop of the diastolic portion. Experimental design schematic with black block indicating data acquisition is below each corresponding graph. ^*^P < 0.05 vs. corresponding baseline.

Compared to baseline in normal animals, 5 weeks of renoprival hypertension (RW) resulted in pronounced cardiac diastolic dysfunction as assessed by echocardiography (decreased E/A ratio; prolonged isovolumetric relaxation time (IVRT), but a minimal effect on systolic function (ejection fraction, EF) (Table 5 and Figure 3A). Invasive (terminal) assessments further demonstrated diastolic dysfunction; elevated filling pressures (LV-EDP), decreased linear slope of the EDPVR and slowed relaxation (Tau), and preserved systolic function (ESPVR and PRSW) (Table 6). It should be noted that since HR was different than normals, the corrected Taus (represented as a % of the R-R interval, Tau_c_) were also evaluated (Table 6). In this setting, CXL-1020 enhanced both diastolic and systolic performance (Table 6, Figure 3A, and represented in Figure 3B). The magnitude of the CXL-1020 mediated improvement in the slope of the EDPVR of RW ventricles was significantly greater than the effects seen in normal animals (Table 6).

**Table 5:** Effect of CXL-1020 on LV on systolic and diastolic functional indices via an echocardiographic evaluation in anesthetized RW and ISO rat models.

**Parameters**	**Model/Timepoint**	**Value ± SEM**	**% Δ from baseline**
EF	Baseline	74 ± 2	–
	RW−2 week	76 ± 1	3 ± 1
	RW−4 week	75 ± 1	2 ± 1
	ISO−4 week	65 ± 1	−12 ± 2^*^
E/A ratio	Baseline	2.1 ± 0.1	–
	RW−2 week	1.9 ± 0.1	−12 ± 3^*^
	RW−4 week	1.7 ± 0.1	−23 ± 2^*^
	ISO−4 week	1.35 ± 0.03	−37 ± 1^*^
IVRT (ms)	Baseline	25 ± 1	–
	RW−2 week	34 ± 1	33 ± 4^*^
	RW−4 week	38 ± 1	50 ± 4^*^
	ISO−4 week	47 ± 3	86 ± 10^*^

*Summary data as values are mean ± standard error of the mean and % change from baseline; n = 6–15*.

* *P < 0.05 vs. baseline*.

**Table 6:** Cardiovascular activity of CXL-1020 in instrumented anesthetized RW and ISO rats.

**Parameters**	**Model**	**Baseline**	**CXL-1020**	**% Δ from baseline**
HR (bpm)	Normal	352 ± 16	353 ± 17	0 ± 1
	RW	389 ± 9	390 ± 9	0 ± 1
	ISO	324 ± 10	324 ± 14	0 ± 2
MAP (mmHg)	Normal	128 ± 5	95 ± 5^*^	−26 ± 2
	RW	141 ± 5	97 ± 6^*^	−23 ± 4
	ISO	132 ± 4	97 ± 6^*^	−26 ± 4
LV–EDP (mmHg)	Normal	4.1 ± 1.1	2.4 ± 1.4^*^	−60 ± 13
	RW	15.1 ± 2.0	9.7 ± 2.0^*^	−40 ± 9
	ISO	14.1 ± 1.6	7.8 ± 2.7^*^	−53 ± 15
ESPVR (mmHg/RVU)	Normal	19.5 ± 1.7	26.1 ± 1.1^*^	37 ± 8
	RW	20.4 ± 1.4	26.3 ± 1.9^*^	29 ± 5
	ISO	16.3 ± 2.2	20.4 ± 2.5^*^	26 ± 5
PRSW (mmHg)	Normal	42 ± 1	53 ± 1^*^	26 ± 1
	RW	44 ± 2	59 ± 4^*^	33 ± 6
	ISO	32 ± 1	45 ± 1^*^	40 ± 5^#^
EDPVR (mmHg/RVU)	Normal	1.4 ± 0.1	1.0 ± 0.1^*^	−29 ± 5
	RW	2.8 ± 0.5	1.2 ± 0.3^*^	−60 ± 5^#^
	ISO	4.3 ± 0.6	1.6 ± 0.3^*^	−63 ± 2^#^
LV–Tau (ms)	Normal	8.9 ± 0.5	7.6 ± 0.4^*^	−14 ± 1
	RW	9.9 ± 0.3	8.3 ± 0.3^*^	−16 ± 2
	ISO	14.3 ± 1.0	10.5 ± 0.8^*^	−27 ± 1^#^
LV–Tau_c_ (%)	Normal	5.4 ± 0.2	4.7 ± 0.1^*^	−12 ± 1
	RW	6.4 ± 0.3	5.4 ± 0.2^*^	−16 ± 1
	ISO	7.7 ± 0.3	5.6 ± 0.2^*^	−27 ± 1^#^

*Changes in systemic and LV hemodynamics indices, and LV mechanical parameters were measured at baseline, and following 30 min of continuous IV infusion of CXL-1020 (100 μg/kg/min, n = 7 Normal, n = 6 ISO, n = 7 RW). Summary data as values are mean ± standard error of the mean and % change from baseline*.

* *P < 0.05 vs. baseline*,

# *P < 0.05 vs. corresponding normal*.

The ISO rats also exhibited cardiac dysfunction as assessed by echocardiography (decreased E/A ratio, prolonged IVRT with slight reduction in EF), but more than RW (Figure 3A). In agreement, invasive assessments demonstrated mild systolic dysfunction (evidenced by depressed ESPVR and PRSW) and pronounced diastolic dysfunction (evidenced by elevated LV-EDP, EDPVR, and Tau) (Table 6). In the ISO model, CXL-1020 significantly enhanced both diastolic and systolic performance (Table 6 and Figures 3D,E) compared to baseline. The magnitude of the CXL-1020 mediated decrease in MAP (% decrease) was similar in ISO and in normal rats (Table 6). However, CXL-1020-induced improvements in load-independent diastolic (Tau, EDPVR) and systolic (PRSW) function were significantly greater in the ISO rats (Table 6) than normals. Taken together, these results demonstrate that CXL-1020 not only remains efficacious in the setting of diastolic dysfunction, but also that its salutary effects were enhanced in experimental dysfunctional myocardium. These results may be related in part to HNO's known mechanism of improving Ca^2+^ cycling.

**Figure 4:**
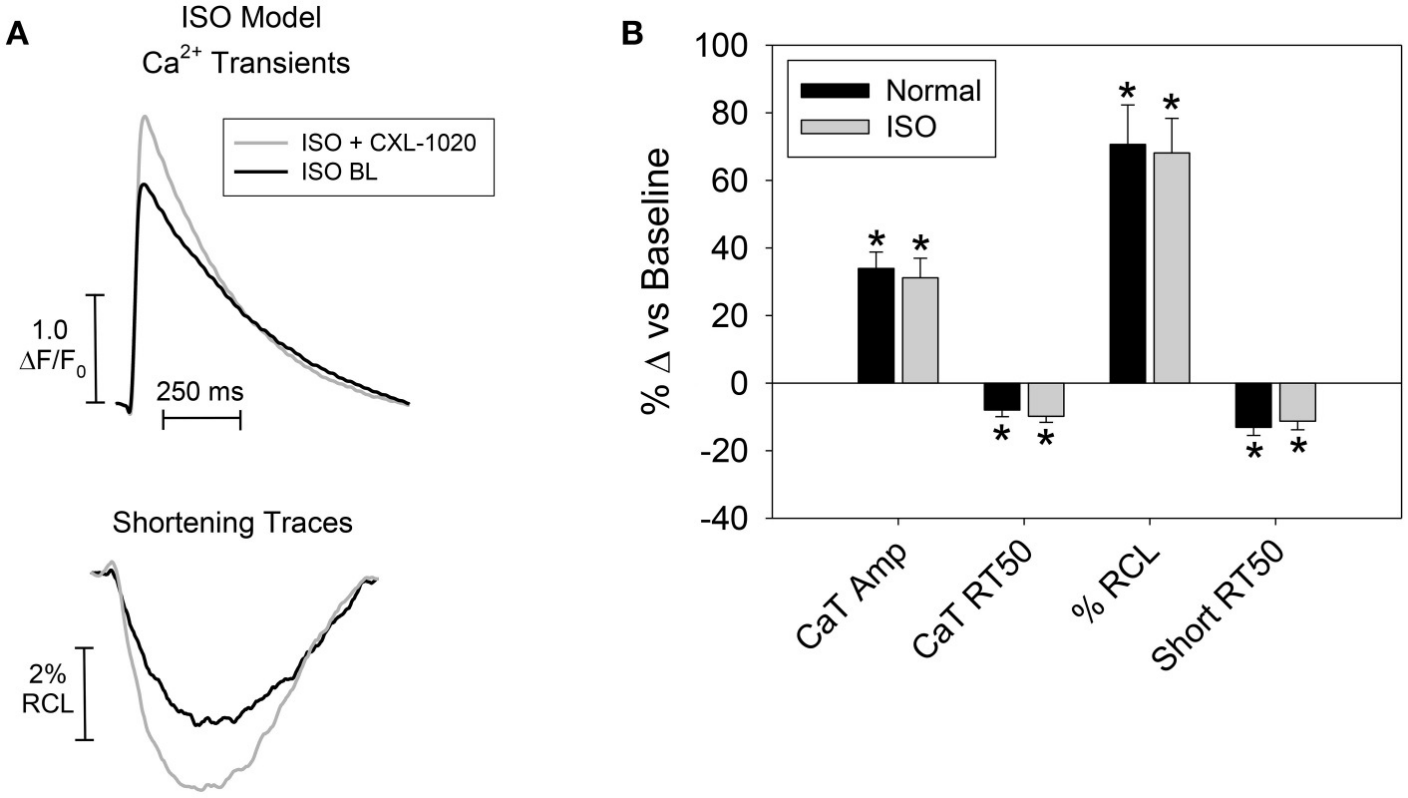
Effect of CXL-1020 on isolated cardiomyocyte function in the ISO rat model. **(A)** Representative trace of Ca^2+^ transients (CaT) and myocyte shortening (as the % resting cell length (RCL)). **(B)** Summary data as % change from baseline of contractile changes (*n* = 14 cells/7 hearts) (CaT and shortening amplitude) and relaxation (CaT and relengthening RT_50_), ^*^P < 0.05 vs. corresponding baseline.

CXL-1020's effects on contraction and relaxation of cardiomyocytes isolated from ISO rats and normal rats were compared (Figure 4 and following results). ISO myocytes had depressed contraction [Ca^2+^ Transient amplitudes (2.0 ± 0.2 vs. 3.1 ± 0.2 ΔF/F_o_; *n* = 31–50 cells/8–9 hearts, ^*^P < 0.05); and reduced shortening amplitudes (1.5 ± 0.3 vs. 2.2 ± 0.2%RCL, ^*^P < 0.05)], and slowed relaxation [Ca^2+^ Transient RT_50_ (235 ± 9 vs. 213 ± 6 ms, ^*^P < 0.05), and prolonged relengthening RT_50_ (312 ± 9 vs. 282 ± 9 ms, ^*^P < 0.05)]. The RT_50_ is the time for Ca^2+^ Transient/myocyte to decline/relengthen to 50% of its max amplitude. Although at a different starting point (i.e., depressed myocyte function in ISO, see values in the text above) in this isolated cardiomyocyte setting, CXL-1020 (50 μM) enhanced myocyte contraction and accelerated relaxation (representative in Figure 4A) and to the same extent in ISO as in normal (shown as % change from baseline) (Figure 4B).

### CXL-1020 is more efficacious than milrinone in rat models of heart failure and does not effect heart rate or rhythm

**Figure 5:**
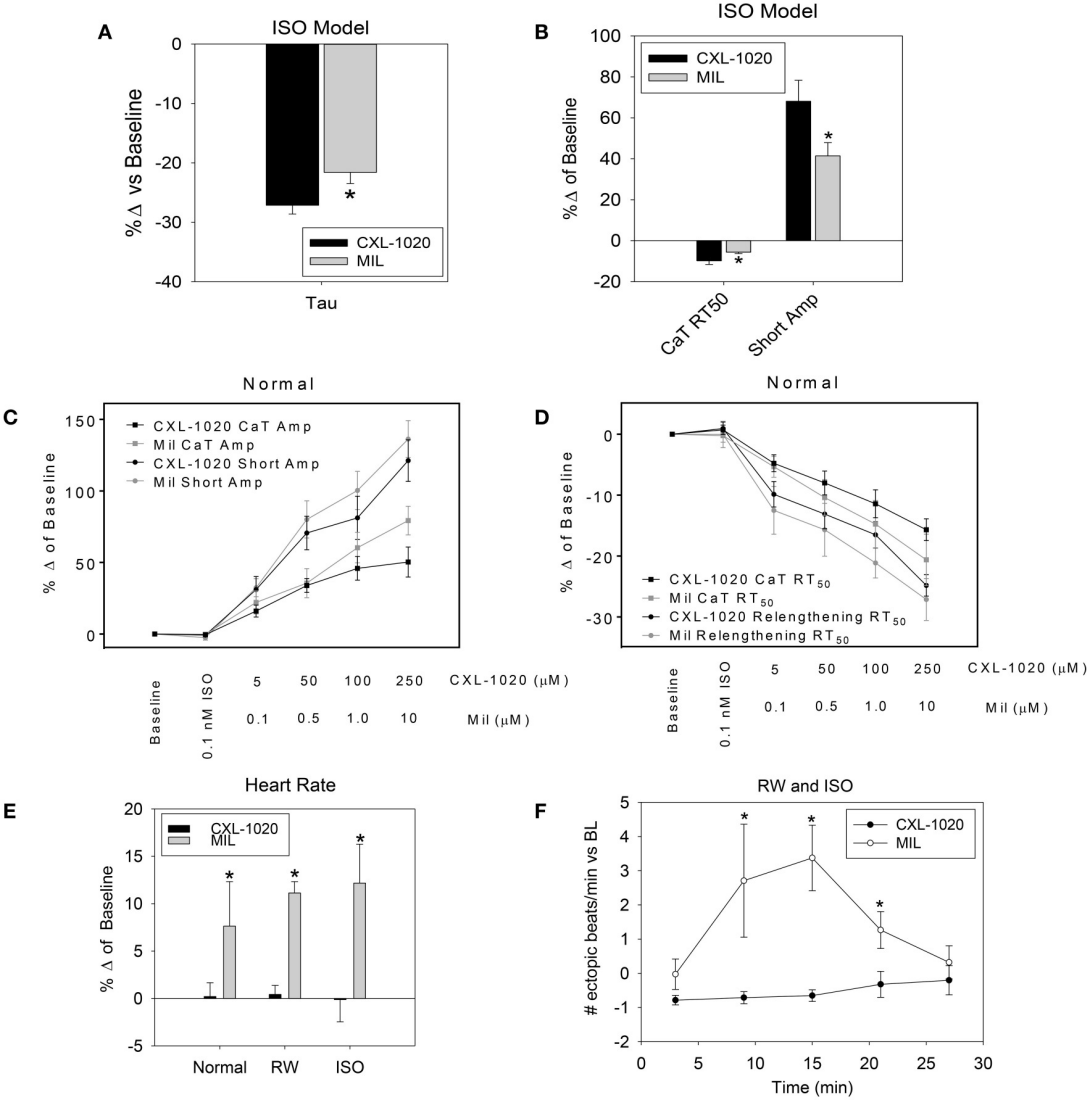
Comparison of CXL-1020 and MIL effects on systemic and LV hemodynamic indices in instrumented anesthetized rats and cardiomyocyte function. Summary data comparing as % change from baseline of **(A)** Tau (*n* = 6) and **(B)** myocyte function (*n* = 14–17 cells/7–8 hearts) in ISO rats. **(C)** Summary data as % change from baseline of a dose response of CXL-1020 and MIL in normal rats on contractile changes (CaT and shortening amplitude) and **(D)** relaxation (CaT and relengthening RT50), 7–25 cells/5–8 hearts. **(E)** Summary data as % change from baseline of CXL-1020 and MIL on heart rate in normal, RW and ISO rats (*n* = 5–7). **(F)** The time course of ectopic beats/per minute from the LV-pressure curve during IV infusion (*n* = 12–13). ^*^P < 0.05 vs CXL-1020.

To further illuminate the hemodynamic efficacy and safety of HNO prodrug CXL-1020, it was compared to the PDE3A inhibitor milrinone (MIL), a clinically relevant drug that has a similar lusitropic inodilator profile, in the *in vivo* and *in vitro* preparations described above (see Table 7 for aggregate results in normals). Both CXL-1020 and MIL enhanced function (EDPVR, Tau) in the diastolic disease models to a greater extent than in normal animals [Table 6 (CXL-1020) and Table 8 (Mil)]. CXL-1020 and MIL demonstrated similar enhancements in contractility (PRSW), linear slope of the EDPVR, and PVA at doses that elicited a similar blood pressure response (MAP) in normal rats (Table 6 (CXL-1020) and Table 8 (Mil)]. However, CXL-1020 had a greater effect than MIL on ventricular relaxation (Tau) in ISO rats (Figure 5A). Consistent with the *in vivo* result, CXL-1020 produced a greater acceleration of the Ca^2+^ Transient RT_50_ and increase in shortening amplitude than MIL in isolated cardiomyocytes (Figure 5B) when assayed at concentrations that elicited similar functional responses in normal myocytes (Figures 5C,D). These results suggest that CXL-1020 is more effective at cycling intracellular Ca^2+^ to enhance myocyte contraction and relaxation than MIL in a setting of chronic β-AR stimulation/diastolic dysfunction. Notably, CXL-1020 had a neutral effect on HR while MIL triggered significant cardioacceleration, consistent with its well-documented positive chronotropic effect (Brunkhorst et al., 1989) (Table 8). The positive chronotropy induced by MIL was even more pronounced in RW and ISO rats (Figure 5E) than in normal animals. Furthermore, MIL, but not CXL-1020 infusion, as shown in a time course during infusion (Figure 5F), also increased the frequency of ectopic beats in both cardiac dysfunctional rats (CXL-1020; normal: −0.1 ± 0.1, RW/ISO: −0.6 ± 0.5, MIL; normal: 0.4 ± 0.3, RW/ISO: 2.1 ± 1.1 # of ectopic beats/min vs. baseline). RW and ISO rats had a higher incidence of ectopic beats (prior to dosing) than normal rats (normal: 0.2 ± 0.2; RW: 0.6 ± 0.1; ISO: 1.1 ± 0.8 # of ectopic beats/min). Thus, under conditions in which CXL-1020 and MIL produced similar hemodynamic and functional improvements, CXL-1020 administration lacked both the chronotropic and myocardial irritability associated with MIL, suggesting a reduced risk for arrhythmic events.

**Table 7:** Cardiovascular activity of Milrinone in instrumented anesthetized normal rats.

**Parameters**	**Normal**		
	**Baseline**	**Milrinone**	**% Δ from baseline**
HR (bpm)	353 ± 9	376 ± 15^*^	6 ± 2
MAP (mmHg)	118 ± 4	79 ± 5^*^	−33 ± 3
LV—ESP (mmHg)	121 ± 3	77 ± 6^*^	−36 ± 4
LV—EDP (mmHg)	5.4 ± 0.5	3.4 ± 0.7^*^	−42 ± 8
LV—EDV (RVU)	17.6 ± 1.1	16.4 ± 1.0^*^	−7 ± 1
SW (mmHg ^*^ RVU ^*^ 10^3^)	1.9 ± 0.3	1.5 ± 0.3^*^	−20 ± 8
PVA (mmHg ^*^ RVU ^*^ 10^3^)	2.3 ± 0.3	1.6 ± 0.3^*^	−32 ± 9
ESPVR (mmHg/RVU)	23.8 ± 1.8	35.0 ± 3.7^*^	45 ± 9
PRSW (mmHg)	45 ± 4	63 ± 6^*^	41 ± 9
EDPVR (mmHg/RVU)	1.8 ± 0.3	1.4 ± 0.3^*^	−19 ± 8
LV—Tau (ms)	8.5 ± 0.4	6.3 ± 0.3^*^	−25 ± 4

*Changes in systemic and LV hemodynamics indices, and LV mechanical and energetic parameters were measured at baseline, and following at least 30 min of continuous IV infusion of Milrinone (10 μg/kg/min, n = 12). Summary data as values are mean ± standard error of the mean and % change from baseline*.

* *P < 0.05 vs. baseline*.

**Table 8:** Cardiovascular activity of Milrinone in instrumented anesthetized RW and ISO rats.

**Parameters**	**Model**	**Baseline**	**Milrinone**	**% Δ from baseline**
HR (bpm)	Normal	366 ± 15	397 ± 29	9 ± 5
	RW	377 ± 11	419 ± 14^*^	11 ± 1
	ISO	340 ± 17	380 ± 20^*^	12 ± 4
MAP (mmHg)	Normal	119 ± 4	80 ± 7^*^	−28 ± 5
	RW	147 ± 4	104 ± 7^*^	−20 ± 4
	ISO	129 ± 5	104 ± 7^*^	−19 ± 3
LV—EDP (mmHg)	Normal	4.2 ± 0.3	2.1 ± 0.4^*^	−54 ± 9
	RW	13.3 ± 2.2	9.0 ± 1.0^*^	−30 ± 7
	ISO	10.3 ± 2.6	5.3 ± 2.2^*^	−56 ± 9
ESPVR (mmHg/RVU)	Normal	19.7 ± 2.6	25.1 ± 3.3^*^	27 ± 5
	RW	19.6 ± 1.5	25.7 ± 1.8^*^	32 ± 6
	ISO	15.8 ± 1.6	20.1 ± 1.7^*^	29 ± 4
PRSW (mmHg)	Normal	42 ± 2	59 ± 5^*^	33 ± 9
	RW	43 ± 2	54 ± 2^*^	26 ± 3
	ISO	30 ± 1	43 ± 1^*^	46 ± 6
EDPVR (mmHg/RVU)	Normal	1.5 ± 0.3	0.8 ± 0.1^*^	−39 ± 12
	RW	2.2 ± 0.3	1.1 ± 0.2^*^	−51 ± 5
	ISO	3.9 ± 0.2	1.5 ± 0.2^*^	−61 ± 4
LV—Tau (ms)	Normal	8.6 ± 0.3	6.8 ± 0.4^*^	−22 ± 3
	RW	10.2 ± 0.3	7.8 ± 0.2^*^	−23 ± 3
	ISO	12.8 ± 1.1	10.0 ± 0.7^*^	−22 ± 2
LV—Tau_c_ (%)	Normal	5.3 ± 0.2	4.4 ± 0.2^*^	−16 ± 3
	RW	6.4 ± 0.1	5.4 ± 0.1^*^	−16 ± 2
	ISO	7.2 ± 0.3	5.9 ± 0.2^*^	−19 ± 1

*Changes in systemic and LV hemodynamics indices, and LV mechanical and energetic parameters were measured at baseline, and following at least 30 min of continuous IV infusion of Milrinone (10 μg/kg/min, n = 6 normal and n = 6 ISO, n = 6 RW). LV-Tau_c_ is Tau corrected to the R-R interval. Summary data as values are mean ± standard error of the mean and % change from baseline*.

* *P < 0.05 vs. baseline*.

**Figure 6:**
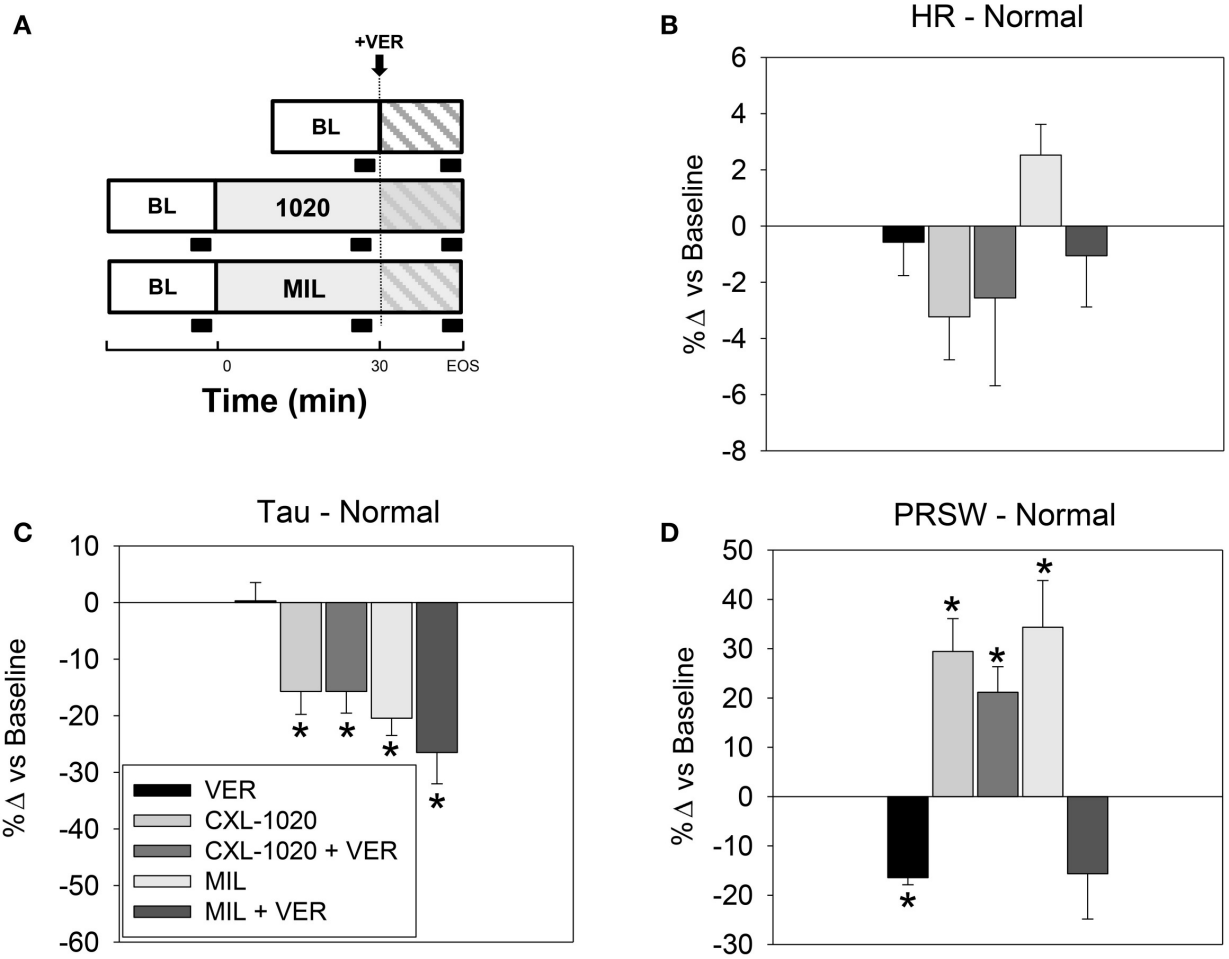
Effects of L-Type Ca^2+^ Channel blockade on systemic and LV hemodynamics indices in instrumented anesthetized normal rats. **(A)** Schematic of the experimental design with black block indicating data acquisition. Summary data as % change vs. baseline of the cardiovascular profiles of verapamil (VER) alone, CXL-1020 (100 μg/kg/min, *n* = 7) and MIL (10 μg/kg/min, *n* = 7) with and without VER on **(B)** heart rate, **(C)** Tau, and **(D)** preload recruitable stroke work. ^*^P < 0.05 vs. corresponding baseline.

The role of L-type Ca^2+^ channel in mediating the effects of MIL vs. CXL-1020 was evaluated by selectively antagonizing that current *in vivo* with verapamil (VER) (see schematic of the experimental design with black block indicating data acquisition in Figure 6A). VER alone had no effect on HR or Tau but decreased PRSW, consistent with its documented vasoactive and negative inotropic profile (Figures 6B–D, Table 9, and Caruana et al., 1987). In the setting of L-type Ca^2+^ blockade, the positive inotropic effect of CXL-1020 was preserved (minimally attenuated) while that of MIL was abolished (Table 9 and Figure 6D). These results confirm the conclusions of previous *in vitro* studies that HNO does not affect L-type Ca^2+^ channel function (Kohr et al., 2010), and is therefore unlikely to possess the arrhythmogenic risk common to cAMP-dependent inotropes and inodilators such as MIL and DOB (Stump et al., 2000).

**Table 9:** Effects of L-Type Ca^2+^ Channel blockade on systemic and LV hemodynamics indices in instrumented anesthetized normal rats.

**Parameters**	**Drug**	**Normal**		
		**Baseline**	**Drug**	**% Δ from baseline**
LV–Tau (ms)	VER	8.7 ± 0.2	8.7 ± 0.4	0 ± 3
	CXL-1020	8.7 ± 0.5	7.3 ± 0.4	−16 ± 4^*^
	CXL-1020 + VER	–	7.3 ± 0.4	−16 ± 4^*^
	MIL	8.4 ± 0.5	6.6 ± 0.4	−20 ± 3^*^
	MIL + VER	–	6.2 ± 0.6	−26 ± 6^*^
HR (bpm)	VER	365 ± 5	363 ± 6	−1 ± 1
	CXL-1020	358 ± 10	346 ± 8	−3 ± 2
	CXL-1020 + VER	–	347 ± 4	−3 ± 3
	MIL	340 ± 8	349 ± 8	3 ± 1
	MIL + VER	–	340 ± 8	−1 ± 2
MAP (mmHg)	VER	134 ± 3	111 ± 4	−17 ± 2^*^
	CXL-1020	115 ± 6	84 ± 7	−27 ± 4^*^
	CXL-1020 + VER	–	71 ± 3	−37 ± 3^*^
	MIL	117 ± 6	81 ± 6	−31 ± 2^*^
	MIL + VER	–	52 ± 3	−55 ± 1^*^
PRSW (mmHg)	VER	56 ± 5	47 ± 4	−15 ± 4^*^
	CXL-1020	54 ± 5	69 ± 6	29 ± 7^*^
	CXL-1020 + VER	–	64 ± 6	21 ± 5^*^
	MIL	48 ± 7	70 ± 10	34 ± 9^*^
	MIL + VER	–	39 ± 3	−16 ± 9

*Changes were measured at baseline, and following 30 min of continuous IV infusion of verapamil (VER) alone, CXL-1020 (100 μg/kg/min, n = 7) and MIL (10 μg/kg/min, n = 7) with and without VER*,

* *P < 0.05 vs. corresponding baseline*.

## Discussion

The present study is the first to thoroughly evaluate the cardiovascular activity of CXL-1020 in rats. These integrative experiments extend previous studies in humans and other species to confirm that CXL-1020 behaves as a load-independent inotrope/lusitrope, improving Ca^2+^ handling without involvement of the β-adrenergic signaling pathway or LTCC. More importantly these studies demonstrated the beneficial functional effects of CXL-1020 in well-established rat models of chronic cardiac dysfunction with abnormal relaxation (hypertension secondary to renal-wrapping and chronic beta-adrenergic stimulation). In the setting of impaired diastole, the beneficial effects of CXL-1020 were not only greater than observed with the cAMP-dependent inotrope MIL, but more importantly, free of chronotropic and pro-arrhythmic liabilities.

### HNO pharmacology ameliorates systolic and diastolic dysfunction

Previous studies have demonstrated the positive inotropic and lusitropic effects of HNO, in normal dogs (Kohr et al., 2010) and dog systolic heart failure models (Sabbah et al., 2013). In conscious normal dogs, HNO increased myocardial contractility (both load-dependent and independent indices) and decreased Tau, indicating improvement of both cardiac systolic and diastolic function. Arterial and LV pressure, as well as EDV were reduced. Although arterial pressures were decreased, HR and calculated systemic vascular resistance (SVR) were unchanged, indicating that cardiac output (CO) was reduced in these euvolemic animals. In the conscious tachypacing-induced heart failure model, the effects of HNO treatment on diastolic and systolic function were similar, however, EDV was not reduced as much, CO was increased, and SVR was decreased.

In the present study, we evaluated the cardiovascular effects of HNO, using CXL-1020, in closed-chest anesthetized rats with normal myocardium, with ISO-induced systolic and diastolic dysfunction, and RW-induced diastolic dysfunction. CXL-1020 enhanced inotropy and lusitropy and caused vasodilation in these rat models. However, the relative magnitude of the effects on inotropy vs vasodilation was different than observed in dogs. For example, at CXL-1020 doses that elicited similar improvements in systolic function (ESPVR) (26% in ISO rats and ~30% in tachypaced dogs), the reduction in MAP in the ISO rats (~−25%) was significantly greater than the reduction in MAP in the dogs (~−10%). These results demonstrate that the ratio of inotropy to vasodilation in the rat is lower than in the dog. This may be, in part, due to the very high efficiency of cardiac Ca^2+^ cycling in rats in which ~97% of intracellular Ca^2+^ is re-sequestered back into the SR, compared to ~75% in dogs. Despite these minor differences, the hemodynamic profile produced by HNO is similar across species [rat, dog, mouse (unpublished), sheep (unpublished), and human (Paolocci et al., 2001, 2003; Sabbah et al., 2013], consistent with the growing body of data suggesting that HNO is an endogenous signaling molecule.

Approximately 50% of patients admitted to the hospital for acute heart failure suffer from diastolic dysfunction (HFpEF) (Sabbah et al., 2013). Although the underlying causes are not completely understood, evidence suggests that abnormal Ca^2+^ handling and PKA/PKG signaling are involved (Yancy et al., 2006). There are no effective therapies for HFpEF, however, several drugs targeting the renin-angiotensin system are currently in HFpEF clinical trials. The effectiveness of CXL-1020 in improving diastolic dysfunction was evaluated here in two rat models. The chronic ISO model is a model of mild systolic dysfunction and more significant diastolic dysfunction due to sub-endocardial fibrosis and slowed SR Ca^2+^ uptake (decreased SERCA2a and PLB Ser16 phosphorylation (PKA site) (Zile et al., 2004; Martos et al., 2007). The present study demonstrated that CXL-1020 improved Ca^2+^ cycling *in vitro* by increasing the rate of Ca^2+^ decline in myocytes (Figures 4A,B) and *in vivo* by decreasing the EDPVR slope (Table 6 and Figure 3). In the bilateral RW model, renoprival hypertension induced pronounced diastolic abnormalities with minimal systolic impairment (Table 5, Figure 3A, and Paolocci et al., 2001; Yancy et al., 2006). In this model, the positive lusitropic actions of CXL-1020 were preserved and it produced an even larger improvement in the EDPVR slope compared to normal rats (EDPVR: −60 vs. −29% *P* < 0.05). This enhanced efficacy in the disease state was not observed in published studies in the conscious tachypaced HF canine model. Collectively, the results in the present study demonstrate the potential of HNO for the treatment of acute clinical diastolic heart failure.

### HNO provides enhanced effectiveness without the chronotropy or increased ectopy associated with established cardioactive agents

The cardiovascular profile of HNO in rats, dogs, and humans is a uniquely attractive profile for the treatment of myocardial systolic and diastolic dysfunction. The ability of HNO to directly enhance cardiac function in addition to vasodilation differentiates it from pure vasodilators (SNP, Table 3 and Figure 1G). The lack of chronotropy and improvement in PVA also differentiates HNO from the cAMP-mediated inotropes/inodilators (i.e., MIL and DOB, Table 3 and Figure 5E).

It should be noted that changes in heart rate, as observed in with MIL administration and diastolic dysfunction model development, can alter the measurement of diastolic function. As HR increases, there is less time for the ventricle to fill and therefore can artificially change diastolic parameters; specifically, the Tau of relaxation (an index of myocardial relaxation and a surrogate for SR Ca^2+^ uptake). The corrected Tau (% of R-R interval) was added due to the disparity between baselines HR between models (i.e., normal, ISO and RW). Elevated HR changes will also reduce the end diastolic volume, shift the LV end diastolic pressure and the EDPVR (Aroesty et al., 1985; Miura et al., 1994). However, the changes observed during this study were minor; only 3–10% off controls and should not affect the diastolic measurements.

In normal rats, the reduction of Tau and myocyte Ca^2+^ transient RT_50_ were greater for MIL compared to CXL-1020. However, in ISO rats, CXL-1020 treatment resulted in faster Ca^2+^ kinetics than MIL (Tau:−27 vs. −22 of BL, *P* < 0.05 and Ca^2+^ transient RT_50_: −9.8 vs. −5.6% of BL, *P* < 0.05) (Figures 5A,B). This enhanced efficacy of CXL-1020 in the disease state may be due to the difference in signaling pathways for HNO and MIL. In the chronic ISO model (Hart et al., 2001), as in patients with HF, elevated circulating catecholamines and sustained catecholamine signaling through the β-AR/cAMP/PKA pathway results in a downregulation of β-AR expression and blunted responses to cardioactive drugs that work through cAMP (i.e., MIL and DOB).

HNO improves cardiac function by enhancing Ca^2+^ cycling and myofilament contractility without affecting intracellular Ca^2+^ content, or LTCC currents. This LTCC-independent mechanism of action has been demonstrated previously *in vitro* (Iaccarino et al., 1998) and is demonstrated *in vivo* in the present study in which VER had a minimal effect on the cardiovascular profile of CXL-1020 (Table 9 and Figure 6). This unique cAMP/LTCC-independent mechanism of enhancing electrocardiogram may partly explain why HNO does not increase the incidence of background ectopy (Figure 5F) or reduce the threshold for triggered arrhythmias (Kohr et al., 2010). The cAMP-mediated agents (MIL, DOB) have proven to be positive chronotropes and increase the risk of arrhythmogenicity, due in part to enhanced extracellular Ca^2+^ influx through the LTCC which increases the probability of spontaneous arrhythmias associated with abnormal Ca^2+^-induced Ca^2+^-release events (Sabbah et al., 2013). Consistent with this, administration of MIL, but not CXL-1020, increased the number of LV ectopic beats (Figure 5F). As the open probability of these Ca^2+^ channels is increased in human failing cardiomyocytes, additional activation potentiates the risk of arrhythmia (January and Fozzard, 1988; Tweedie et al., 2000; Wang et al., 2008). Thus, not only does the cardiovascular profile of HNO suggest benefit in the treatment of ADHF, but also suggests a more favorable cardiovascular electrophysiological safety profile than other established inotropes/inodilators.

### Pharmacodynamic interactions between CXL-1020 and other cardioactive drugs are complementary and consistent with the HNO molecular mechanism of action

Unlike cAMP-dependent positive inotropes and inodilators, the preclinical pharmacology of HNO was shown as non-interactive with concomitant β-blocker therapy (Figure 2B) (Schroder et al., 1998). CXL-1020's ability to reduce arterial pressure, preserve PVA, and enhance cardiac contractility and relaxation was maintained when co-administered with either the ACE inhibitor enalapril or the β-blocker metoprolol (Figures 2A,B). In both settings, there was a mild augmentation in vasodilation compared to their independent effects. The lack of pharmacodynamic drug-drug interaction suggests that in the clinical setting, HNO therapy could be used concomitantly with β-blocker and/or ACE inhibitor drugs in patients that present with ADHF. Continued administration of concomitant background HF medications during hospitalization may streamline the transition from the inpatient to outpatient setting. In addition, the complementary pharmacologic effects suggest that HNO could augment β-blocker and ACE inhibitor effects in the treatment of heart failure. Further experimental and clinical drug-drug interaction evaluations must await the availability of oral HNO prodrugs. Although the combination of an HNO prodrug and a β-AR agonist is not likely to be used clinically, we investigated the effect of CXL-1020 given concomitantly with DOB to further explore whether CXL-1020's mechanism of enhancing cardiac function was independent of β-adrenergic stimulation. In agreement with previously published data in dogs (Paolocci et al., 2003), the combined response of co-administration of CXL-1020 and DOB was additive (Figure 2B and Table 1 vs. Table 3), demonstrating that the CXL-1020 mechanism of action is independent of the β-adrenergic signaling pathway. Since heart failure patients often present with down-regulated β-adrenergic pathway function, a therapeutic that works independently of the β-adrenergic pathway should maintain efficacy, whereas therapies targeting key proteins in the β-adrenergic signaling pathway (MIL-cAMP/PKA) will be less effective. This was previously demonstrated in myocytes from mice that underwent transverse aortic constriction (TAC) (Paolocci et al., 2003; Tocchetti et al., 2007) and again in the present study comparing the effectiveness of CXL-1020 and MIL in ISO rats. In summary, the cAMP/LTCC-independent mechanism of action of HNO, as predicted by molecular and cellular studies, is consistent with the pharmacodynamic drug interactions of CXL-1020 with other cardioactive agents *in vivo*.

## Conclusion

This study demonstrated that the cardiovascular profile of HNO delivered by CXL-1020 in normal rats is similar to the previously published data with Angeli's salt and CXL-1020 in dogs, and demonstrated significant efficacy in models of cardiac diastolic dysfunction. Additionally, these studies demonstrate a superior hemodynamic profile compared with established inotropes/inodilators in two cardiac diastolic dysfunction models, and further elucidate the distinction between the mechanism of action of HNO and these cAMP-dependent inotropes. These studies also demonstrate that CXL-1020 pharmacology is complementary to common background HF therapeutic agents. Early clinical studies have indicated that HNO could be a promising new therapy for systolic HF. The data in the present study suggest that further evaluation of HNO as a therapy for HFpEF is warranted.

## Ethics statement

The procedures used in this manuscript were reviewed and approved by QTest Labs IACUC for compliance with regulations and current accepted practices. The number of animals used in this study aimed to be the minimum number necessary for the evaluation of the results. The procedures are meant to ensure that the animals' exposure to pain and distress are minimized. All animals will be humanely euthanatized in accordance with accepted American Veterinary Medical Association (AVMA) (AVMA Guidelines on Euthanasia, 2013 Edition).

## Disclosures

JR and JH are current employees of Cardioxyl Pharmaceuticals. RM is a prior employee of Cardioxyl Pharmaceuticals.

## Author contributions

SR and CLdR: preformed research, analyzed data, contributed to writing the manuscript. YU: preformed research, analyzed data. RM: contributed to study design. RH, JH, MZ, and JR: contributed to study design, contributed to writing the manuscript.

### Conflict of interest statement

The authors declare that this study received funding from Cardioxyl Pharmaceuticals, Inc. The funder and employees (JR, JH, and RM) were all involved in the study design. JR and JH also contributed to writing the manuscript. QTest Labs performed the experiments under contracts (funding) established between QTest Labs and Cardioxyl Pharmaceuticals, Inc. The other authors declare that the research was conducted in the absence of any commercial or financial relationships that could be construed as a potential conflict of interest.
